# Within-session decrement of the emission of licking bursts following reward devaluation in rats licking for sucrose

**DOI:** 10.1371/journal.pone.0177705

**Published:** 2017-05-11

**Authors:** Paolo S. D'Aquila, Adriana Galistu

**Affiliations:** Dipartimento di Scienze Biomediche, Università di Sassari, Sassari, Italy; University of Leicester, UNITED KINGDOM

## Abstract

We previously observed that dopamine D2-like receptor blockade in rats licking for sucrose produced a within-session decrement of the emission of licking bursts similar to the effect of either reward devaluation, or neuroleptics, on operant responding for different rewards, which, accordingly, we interpreted as an extinction-like effect. This implies that exposing animals to reward devaluation would result in a drop of burst number taking place only after the contact with the devalued reward. To test this prediction, we compared the difference in the within-session time course of burst number in response to high (10%) *versus* low (2%) concentration sucrose solutions, either in a condition of reward devaluation (exposure to 2% after daily 10%), or in a condition which does not involve changes in the reward value (two groups of subjects each repeatedly exposed to only one of the two concentrations). Reward devaluation resulted in a within-session decrement of the burst number, with the response rate dropping only after the contact with the devalued reward, as predicted. This response pattern was reliably observed only in subjects at their first devaluation experience. In contrast, exposure of separate groups of animals to the two different concentrations yielded lower levels of burst number in the low concentration group apparent since the beginning of the session, as previously observed with dopamine D1-like receptor blockade. These results show that the analysis of burst number, but not of burst size, reveals a specific activation pattern in response to reward devaluation, which differs from the pattern observed comparing the response to two different sucrose concentrations in separate groups of subjects, i.e. in a condition not involving reward devaluation. Finally, the characterisation of the experimental measures of the analysis of licking microstructure in behaviourally (and psychologically) meaningful functional terms, might be relevant for the investigation of the mechanisms underlying behavioural activation and the related evaluation processes.

## Introduction

Licking behaviour in rats ingesting fluids is characterised by the clustering of licks in bursts, i.e. discrete series of licks at the rate of about 5 to 7 licks *per* second, as shown by the early studies by Davis based on the log-survivor analysis of the intervals between successive licks [1]. Burst size, defined as the number of licks *per* burst, along with the initial lick rate, is mainly dependent on the nature and the concentration of tastants in a solution. For example, it was demonstrated that burst size is monotonically related to the concentration of different sugars [2–5]. On the other hand, burst number, which represents the number of times that subjects decide to engage in licking behaviour, is more sensitive to stimuli that do not involve the orosensory contact with the reward, such as post-ingestive cues [2,4,5]. Therefore, the number and the size of licking bursts might be interpreted as measures revealing, respectively, (i) a process of activation of a reward-oriented response, possibly due to the attribution of incentive motivational properties to a reward-related stimulus, and (ii) an evaluation process occurring during the consummatory transaction with the reward, which is possibly related to the experience of pleasure [6–9]. Rats show a high preference for sucrose, the ingestion of which appears to be regulated by both orosensory and post-ingestive mechanisms, which determine the total lick number in a session by primarily influencing size and number of licking bursts [10–14].

We recently suggested that the activation of reward-associated responses depends on dopamine D1-like receptor stimulation, while the level of this activation is updated, or “reboosted”, on the basis of a dopamine D2-like receptor-mediated evaluation process occurring during the consummatory transaction with the reward [8, 9, 15]. This hypothesis rests on the observation that dopamine D2-like receptor antagonists, not only reduce the size of licking bursts for sucrose [3, 8, 16–18] thus mimicking the effect of sucrose dilution [3], but also result in a within session decrement of burst number occurring after the contact with the reward, which we interpreted as an extinction-like effect [8,9], as previously suggested for the effect of either reward devaluation or neuroleptics on instrumental responding for different rewards [19–21]. In contrast, dopamine D1 receptor blockade reduces lick number exclusively by reducing burst number, but has no effect on their size [8, 9, 18, 22–24]. It is worth noting that its effect on the burst number within-session time course is apparent since the very beginning of the sessions, thus differing substantially from the effect of dopamine D2 receptor blockade [8,9]. Consistent results were obtained examining the effect of dopamine D1- and D2-like receptor antagonists in sodium-replete [9, 23–25] and sodium-depleted [23] rats licking for NaCl solutions.

The interpretation of the effect of dopamine D2 receptor blockade on the within-session time course of burst number as an extinction-like effect–which is the main piece of evidence in support of the proposed hypothesis–implies that exposing animals to reward devaluation, obtained by reducing the concentration of a familiar sucrose solution, would also result in a similar response, i.e. a drop of burst number taking place only after the contact with the devalued reward. To our best knowledge, the effect of sucrose dilution on the licking burst number time course was never reported in the literature so far. Indeed, earlier studies investigated the within-session time course of lick number, but not of burst number, after either reward devaluation or neuroleptics, failing to observe such a response pattern [26, 27]. Thus, the aim of this study was to test this prediction.

This study consists into two experiments. Experiment 1 was aimed (i) to the investigation of the effect of reward devaluation, both within subjects and in comparison with a group not subjected to reward devaluation, and (ii) to the exploration of the effects of repeated exposure to reward devaluation. To this end, we examined the effect of sucrose dilution on the licking burst number time course (along with the other microstructural measures of licking behaviour) within 30-min sessions in rats with an experience of daily exposure to a 10% sucrose solution, i.e. in an experimental condition involving reward devaluation. In a first experimental trial, half of the subjects trained to drink a 10% sucrose solution were offered a 2% solution, and the other half were offered the usual 10% solution. A week later, both groups were subjected to reward devaluation. Since subtle differences between the responses of the two groups were noted, we decided to further explore the possible effects of the repeated experience of reward devaluation in a third trial performed a week later.

Experiment 2 aimed (i) to the replication of the main finding from Experiment 1, i.e. the effect of reward devaluation on the within-session burst number time course, and (ii) to provide an experimental condition apt to reveal the difference between the response pattern to 10% vs 2% sucrose concentration solutions, in absence of reward devaluation. In addition, we decided to explore the effect of an upshift in sucrose concentration in the low concentration group. To this end, two groups of rats were allowed daily access to either a high (10%) or a low (2%) concentration sucrose solution. In the course of 19 experimental sessions, the high concentration group was exposed to the 2% concentration solution in 3 devaluation trials 3–4 sessions apart from each other. In the last session, both groups were exposed to the high concentration solution. The comparisons which bear relevance to the aim of this study are: (i) the within-group comparison between the devaluation session with the immediately preceding one in the high concentration group–i.e the effect of reward devaluation–which provides the replication of the findings from Experiment 1, and (ii) the comparison between the high and the low concentration groups in the sessions not involving downshift (or upshift) in sucrose concentration, which provides a comparison between the response to 10% vs 2% sucrose in absence of reward devaluation. It must be noted that this experimental design is the same which is adopted in the investigation of the successive negative contrast effect, which consists in a reduced intake of the low sucrose concentration solution in the high concentration group when subjected to downshift in concentration, compared to the intake of the unshifted low concentration group [28–30]. This effect is revealed by the comparison between the high and the low concentration group response with both exposed to the low concentration solution.

The results show that sucrose dilution results, as predicted, in a within-session decrement of burst number, taking place only after the contact with the reward, as previously shown with dopamine D2-like receptor blockade [8,9], while the different response to high *versus* low concentration sucrose solutions of separate groups of animals yields lower levels of burst number in the low concentration group, which are apparent since the beginning of the session, as we previously observed with dopamine D1-like receptor blockade [8,9].

## Materials and methods

### Subjects

Experimentally naïve male Sprague-Dawley rats (Harlan, Italy) weighing 300–350 g were used as subjects. The animals were housed in groups of two-three per cage in controlled environmental conditions (temperature 22–24°C; humidity 50–60%; light on at 08:00, off at 20:00), with free access to food and water. All the experimental procedures were carried out in accordance with the regulatory requirement of the Italian law (D.L. 116, 1992) and Council Directive 2010/63EU of the European Parliament, and were approved by the Independent Committee of Bioethics for Animal Testing of the University of Sassari (11/11/2013) and authorised by the Ministry of Health, Italy (Authorisation N. 87/2014-B, 12/03/2014). After the experiments the animals were euthanised with pentobarbital sodium.

### Apparatus, microstructural measures and testing conditions

Behavioural testing was carried out using a multistation lick analysis system (Habitest,Coulbourn Instruments, USA) connected to a computer. Rats were individually placed in a Perspex chamber with an opening in the centre of the front wall allowing access to a bottle spout. The recording period started either after the first lick or after 3 min that the animals were placed into the chambers, so that the latency to the first lick had a cut off time of 3 min. The interruptions of a photocell beam by each single tongue movement while licking the spout were recorded, with a temporal resolution to the nearest 50 milliseconds. The raw data were analysed through Graphic State 3.2 software (Coulbourn Instruments, USA) and, besides lick number, the following microstructural measures were obtained: number of bursts, time spent in bursts, latency to the first lick. A burst was defined as a series of licks with pauses no longer than 400 milliseconds (see [8]). Burst size (number of licks *per* burst) and intra-burst lick rate (lick/sec within bursts) were then calculated. The data were collected in time bins of 3 min in sessions of 30 min.

The experiments were performed between 09:00 and 13:00, i.e. during the light phase of the lighting cycle (see [9]). All the experiments were perfomed in non-deprived animals.

### Experimental design and procedures

#### Experiment 1

The subjects (N = 39) were exposed to a 10% sucrose solution in daily sessions of 30 min. On day 6 of the 2nd week, i.e. after 13 days of daily exposure to sucrose, having obtained a reasonably stable baseline, the subjects were divided into two experimental groups, G1 (n = 20) and G2 (n = 19), matched according to the whole session burst size. The successive day, i.e. on day 7 of the second week (Trial 1), G1 was exposed again to the usual 10% solution, while G2 was exposed to a 2% sucrose solution. Daily exposure of the animals to the sucrose solutions was carried on for two more weeks, with both groups being exposed to the 2% solution on day seven of each week (Trial 2 and 3), while receiving the 10% solution all the other days (Table 1).

**Table 1:** Experimental schedules. Experiments 1 (G1, G2) and 2 (High and Low concentration group). T, trial; D1, D2, D3 2%, R1 10% sucrose solution; ↓/↑: downshift/upshift in sucrose concentration.

Group	Experimental schedules						
		↓		↓		↓	↑
G1	10% 13 days	10%T1	10% 6 days	2%T2	10% 6 days	2%T3	
G2	10% 13 days	2%T1	10% 6 days	2%T2	10% 6 days	2%T3	
High G	10% 8 days	D1	10% 4 days	D2	10% 3 days	D3	R1
Low G	2% 8 days	D1	10% 4 days	D2	10% 3 days	D3	R1

#### Experiment 2

The subjects (N = 35) were allocated into two groups, (i) high concentration group (n = 19) and (ii) low concentration group (n = 16), with 30 min daily access to either a 10% or a 2% sucrose solution, respectively (sessions S1-S15). At the 9^th^ (D1, immediately after S8), 14^th^ (D2, immediately after S12) and 18^th^ (D3, immediately after S15) sessions, the high concentration group was exposed to the 2% concentration solution (3 devaluation trials). In the last session (R1), both groups were exposed to the high concentration solution (10%).

### Data analysis

Statistical analysis of all sets of data was performed with ANOVA, by the software Statistica 8.0 (StatSoft Inc.). Post hoc analysis of the main effects was made using the Newman-Keuls multiple comparison test. When a significant interaction between factors was revealed, comparisons were performed by F-test for contrasts.

#### Experiment 1

The analysis of the results included the data from the 3 devaluation sessions (labeled in the figures as session II) along with the data from the previous day (session I), so that each devaluation trial was composed of two sessions, the first with the 10% solution and the second with the 2% solution (with the exception of the 1^st^ trial for G1, with the animals being offered a 10% solution, see above). Lick number and burst number data analysis involved *devaluation* (2 levels, corresponding to the 2 sessions from each trial), *time* (10 levels, corresponding to the within-session 3-min time bins) and *trial* (3 levels) as within-group factors, and (ii) *group* (2 levels, corresponding to G1 and G2) as a between-group factor. Analysis of the data relative to the latency to the 1^st^ lick, and the whole session number of licks *per* burst and intra-burst lick rate data did not involve the factor *time*. Further analyses were performed to explore the possible within-session changes in burst size. Thus, the data relative to the number of lick *per* burst were grouped into three time bins: T1 (including the first three 3-min time bins, from the beginning of the session up to 9 min), T2 (three 3-min time bins from 9 to 18 min) and T3 (four 3-min time bins from 18 min up to the end of the session). A first analysis involved two within-group factors, *time* (with 3 levels corresponding to the three time bins) and *devaluation* (with two levels, see above) and the between-group factor *group* (with two levels). However, the repeated measures ANOVA does not allow empty cells in the data matrix, thus, the data of the subjects failing to perform a single lick burst in a single time bin are not taken into account in the analysis. Since, especially late in the session, a significant number of subjects fail to engage in licking behaviour, in order to have more representative samples, the data relative to each time bin were also analysed independently, with *devaluation* as a within-group factor and *group* as a between-group factor.

#### Experiment 2

Firstly, the data from either the total or the mean values (as appropriate for each experimental measure) from each session were analysed by ANOVA, with *group* as a between-groups factor (with two levels, corresponding to the high and the low concentration groups), and *session* (with 19 levels, corresponding to the experimental sessions, from S1 to R1) as a within-group factor (by sessions analysis). Moreover, the burst number within-session time course data from the first 7 sessions (S1-S7), from the three devaluation trials and from the concentration upshift trial, R1 (each including the devaluation session–or the R1 session–along with the preceding session: S8-D1, S12-D2, S15-D3, D3-R1), were analysed by ANOVA. The analysis of the data from S1 to S7 involved a between-group factor, *group*, with two levels (corresponding to the high and the low concentration groups), and a within-group factor, *time*, with 10 levels (corresponding to the ten 3 min time bins within the 30 min session). The analysis of the data from the devaluation trials and from the concentration upshift trial involved a further factor, *devaluation* (or *upshift*), with two levels (corresponding to the two sessions of the trial). Further analyses were performed on the within-session time course of burst size, by grouping the session data into 3 time bins, as described for Experiment 1. ANOVA of the data relative to the comparison between each devaluation session and the immediately preceding session in the high concentration group–i.e. the investigation of the effects of devaluation–involved two within-group factors: *devaluation* (2 levels) and *time* (3 levels corresponding to the three time bins). ANOVA of the data relative to the comparison between the high and the low concentration groups in each devaluation session–i.e. the investigation of the contrast effect–involved the within-group factor *time* (3 levels) and the between-group factor *group* (2 levels). As in Experiment 1, the data relative to each time bin were also analysed independently, with *devaluation* as a within-group factor to reveal the effect of devaluation in the high concentration group, and a separate analysis with *group* as a between-group factor to reveal the contrast effect in each devaluation session. Finally, ANOVA of the upshift concentration trial (High and Low concentration group at sessions D3 with 2% and R1 with 10%) involved the between-group factor *group* (2 levels) and the within-group factors *upshift* (2 levels) and *time* (3 levels, corresponding to the three time bins). ANOVA of the data from each single time bin involved the between-group factor *group* and the within-group factor *upshift*.

## Results

### Experiment 1

**Fig 1:**
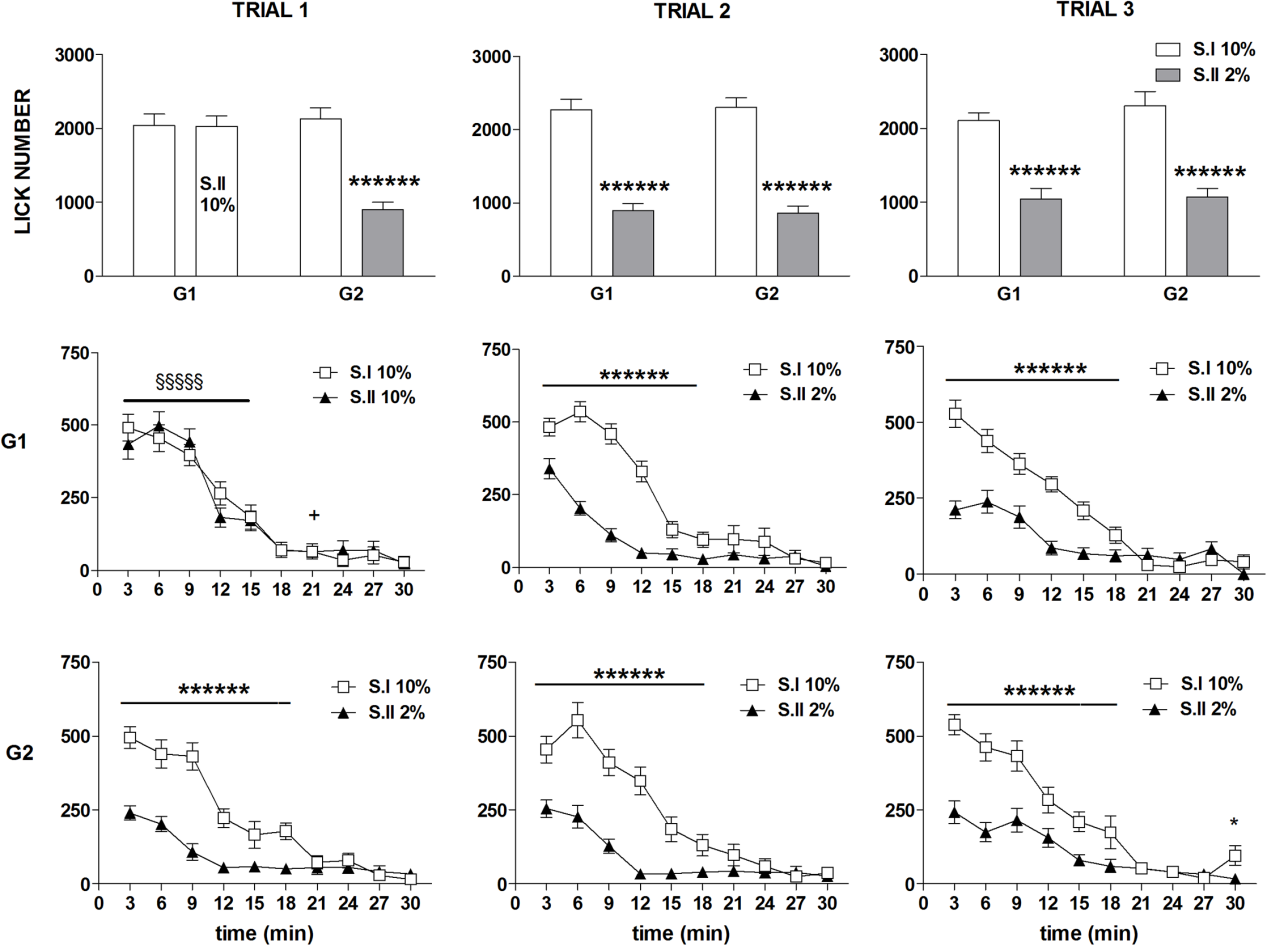
Experiment 1. **Effect of exposure to sucrose dilution on lick number.** Comparison between session I (S.I) and session II (S.II) across the three trials in the groups G1 and G2. Top panels show the total values, mid and bottom panels show the time course of the response in G1 and G2, respectively. Values represent the mean ± S.E.M. from 19–20 subjects. S.I vs S.II in G2: *P<0.05, ******P<10^−6^; G1 vs G2 in S.I: +<0.05; G1 vs G2 in S.II: §§§§§P<10^−5^ (ANOVA followed by F-test for contrasts; straight lines indicate contrasts involving consecutive time points).

ANOVA of lick number data showed a statistically significant effect of *devaluation* [F(1,37) = 293.78, P<10^−6^], and *time* [F(9,333) = 205.71, P<10^−6^], with no statistically significant effects of *trial* [F(2,74) = 1.90, n.s.] and *group* [F(1,37) = 1.28, n.s.]. Moreover, a statistically significant 3 ways interaction between *devaluation*, *trial*, and *group* [F(2,74) = 7.73, P = 0.00089] and a statistically significant 4 ways interaction between *devaluation*, *trial*, *time* and *group* [F(18,663) = 2.53, P = 0.0004] were revealed. Further analyses (F-tests for contrasts) based on the 3 ways interaction showed that, in all the trials and both in G1 and G2, exposure to sucrose dilution resulted in a marked decrease of the total lick number (Fig 1, first row). Comparisons made on the basis of the 4 ways interaction showed that these differences were due to a reduced number of licks from the beginning of the session up to the 18^th^ min in response to the 2% sucrose solution compared to the 10% solution (Fig 1, 2^nd^ and 3^rd^ rows). As for the comparison between G1 and G2 in the first trial, in session I, with both groups being exposed to a 10% sucrose solution, the curves representing the lick number time course are almost superimposable, showing a small but statistically significant difference limited to one time bin (15–18 min). In session II, G2, i.e. the group exposed to the solution at reduced concentration, showed a reduced level of the number of licks since the beginning of the session up to the 15^th^ min compared to G1, which was exposed to the 10% solution (Fig 1, left panels).

**Fig 2:**
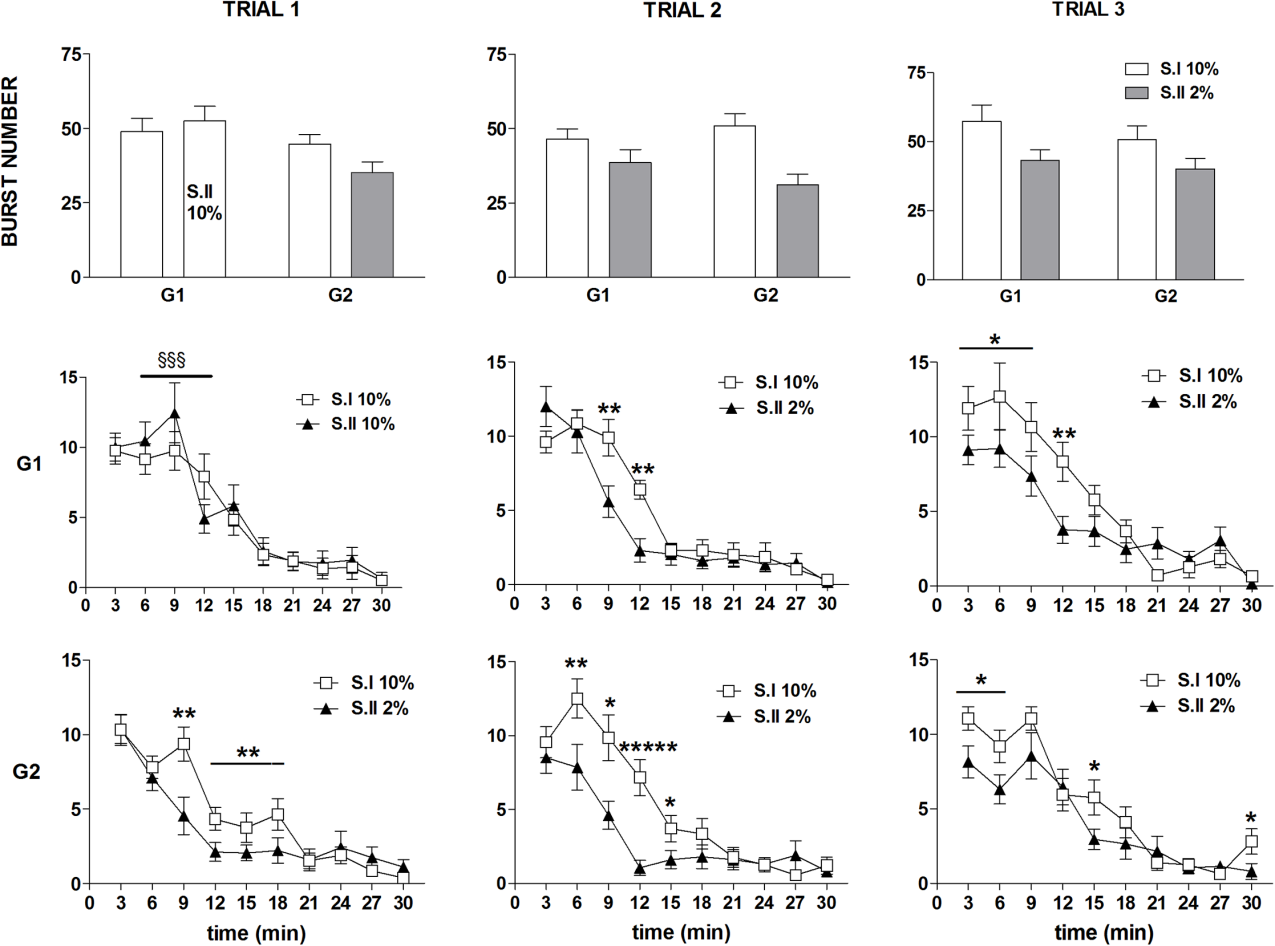
Experiment 1. **Effect of exposure to sucrose dilution on burst number.** Comparison between session I (S.I) and session II (S.II) across the three trials in the groups G1 and G2. Top panels show the total values, mid and bottom panels show the time course of the response in G1 and G2, respectively. Values represent the mean ± S.E.M. from 19–20 subjects. S.I vs S.II in G2: *P<0.05, **P<0.01, *****P<10^−5^; G1 vs G2 in S.II: §§§P<0.001 (ANOVA followed by F-test for contrasts; straight lines indicate contrasts involving consecutive time points).

**Fig 3:**
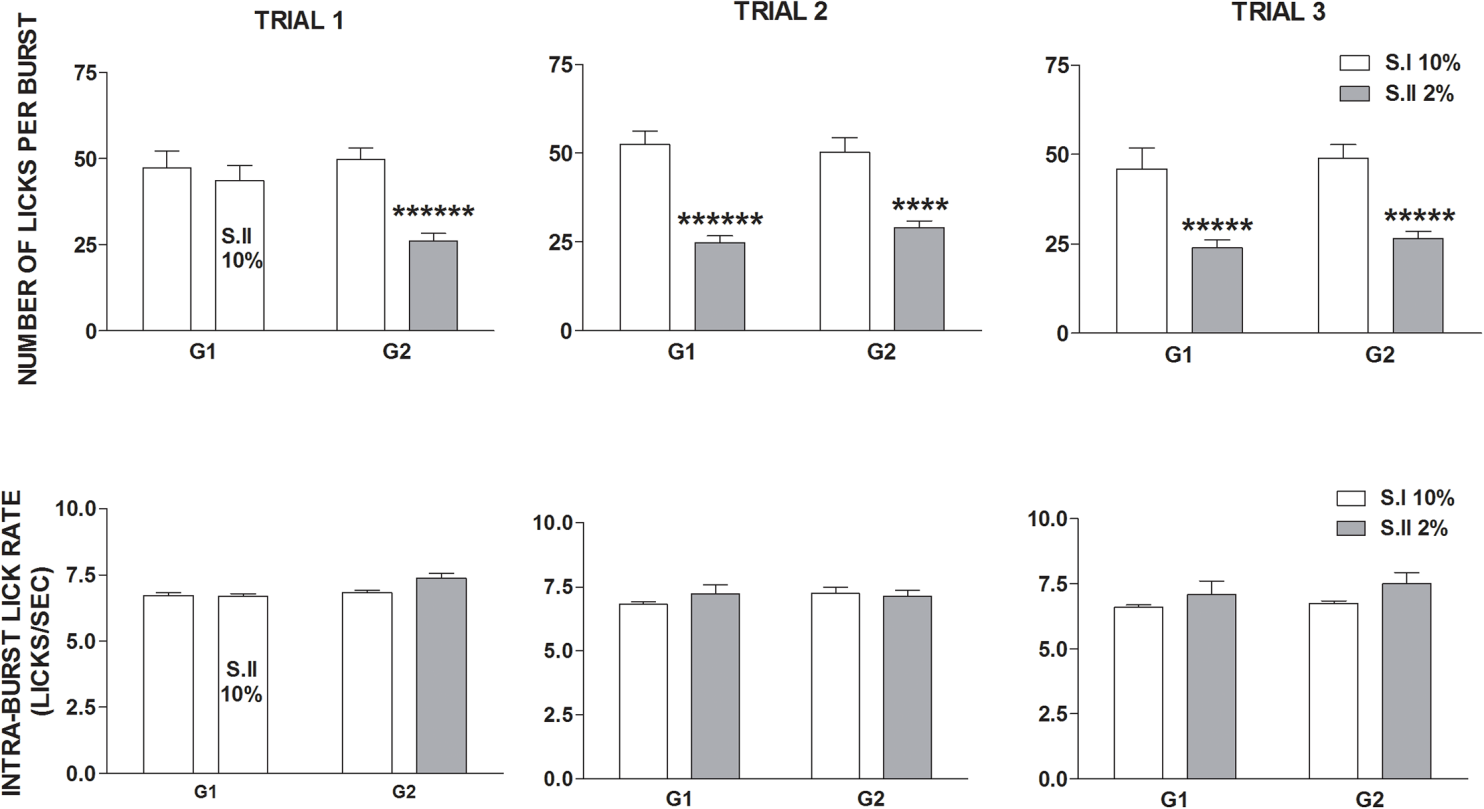
Experiment 1. **Effect of exposure to sucrose dilution on the whole session number of licks *per* burst (top panels) and intra burst-lick rate (bottom panels).** Comparison between session I (S.I) and session II (S.II) across the three trials in the groups G1 and G2. Values represent the mean ± S.E.M. from 19–20 subjects. ****P<10^−4^, *****P<10^−5^, ******P<10^−6^ (ANOVA followed by F-test for contrasts).

ANOVA of burst number data revealed a statistically significant effect of *devaluation* [F(1,37) = 14.17; P = 0.0005], due to the reduced burst number in session II in both groups regardless of trial [*devaluation* x *trial* x *group*: F(2,74) = 1.07; n.s.] (Fig 2, first row). Moreover, a statistically significant effect of the factors *trial* [F(2,74) = 3.70; P = 0.029] and *time* [F(9,333) = 137.82; P<10^−6^] was revealed, with no significant effect of the factor *group* [F(1,37) = 2.74; n.s.]. A more clear picture emerged from the comparisons between the time course data based on the significant 4 ways interaction [F(18,666) = 1.98; P = 0.008] (Fig 3, 2^nd^ and 3^rd^ row). In trial 1, exposure to the diluted solution in G2 (session II) resulted in a within-session decline which resulted in a significant reduction with respect to the response to the 10% solution (session I) from the 3^rd^ to the 6^th^ time bin (Fig 2, 3^rd^ row, left panel). The within session-decline in response to sucrose dilution was apparent also in the comparison between G1 and G2 in session II of the trial I, due to a significant reduction of burst number from the 2^nd^ to the 5^th^ time bin, while a superimposable time course of the responses of the two groups was observed in session I, with both groups exposed to the 10% solution (Fig 2, left panels). In trial 2, a within-session decline of burst number in response to sucrose dilution was observed in both groups. However, in G1, which was at its first exposure to reward devaluation, the difference with respect to the 10% solution was apparent starting from the 3^rd^ time bin, as observed in G2 at its first exposure (trial 1), while in G2, now at its second exposure to devaluation, the difference between the two solutions was anticipated, starting at the 2^nd^ time bin (2^nd^ and 3^rd^ rows mid panels). In trial 3 the difference between the response to the two solutions (session I *vs* session II within each group) was apparent since the very beginning of the session in both groups (Fig 2, 2^nd^ and 3^rd^ rows right panels). The pattern emerging from these results shows a within-session decline of burst number in response to sucrose dilution, which is very clear at the first episode of reward devaluation in both groups, but the reduction of the response level appears to be anticipated following repeated experiences of reward devaluation.

ANOVA of the whole session number of licks *per* burst data showed a statistically significant effect of *devaluation* [F(1,37) = 87.81; P<10^−6^], with no significant effects of *group* [F(1,37) = 0.07; n.s.] and *trial* [F(2,74) = 2.32; n.s.]. Moreover, a statistically significant interaction between *devaluation*, *trial* and *group* was revealed [F(2,74) = 8.26; P = 0.0005]. F-test for contrasts showed a statistically significant reduction of this parameter in response to the 2% sucrose solution, in both groups and in all trials (Fig 3, top panels).

**Fig 4:**
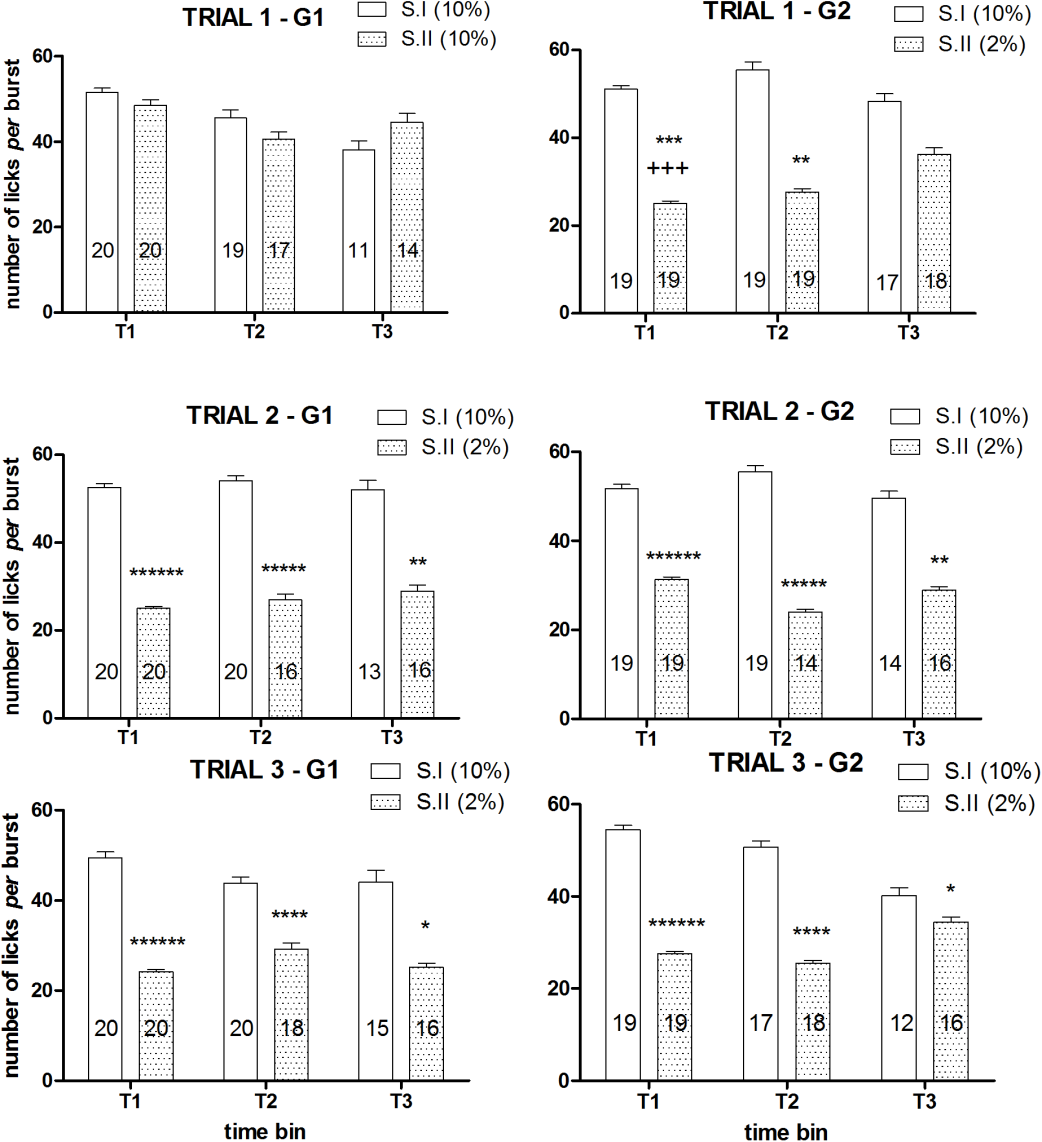
Experiment 1. **Effect of exposure to sucrose dilution on number of licks *per* burst: within-session time course (Experiment 1).** Comparison between session I (S.I) and session II (S.II) across the three trials in the groups G1 (left panels) and G2 (right panels). Values represent mean ± S.E.M. The number of subjects for each mean value is indicated in the relative column. Effect of devaluation: *P<0.05, **P<0.01, ****P<10^−4^, *****P<10^−5^, ******P<10^−6^ (ANOVA, main effect); G2 vs G1: ++P<0.01 (ANOVA followed by Newman-Keuls multiple comparison test).

ANOVA of the within-session time course of number of licks *per* burst of the first devaluation trial showed a significant main effect of the factor *devaluation* [F(1, 23) = 8.58, P = 0.007] and a significant interaction between *devaluation* and *group* [F(1, 23) = 15.86, P = 0.00058], due to a reduced burst size after reward devaluation in the group G2, with no 3 ways interaction between these two factors and *time* [F(2, 46) = 1.62, n.s.]. ANOVA of the data of the first two time bins (T1, T2) revealed a significant interaction between the factors *group* and *devaluation* [T1: F(1, 37) = 17.82, P = 0.00015; T2: F(1, 33) = 8.07, P = 0.0076]. Post-hoc analysis (Newman-Keuls) showed a statistically significant reduction of the number of lick *per* burst in the group G2 in time bins T1 and T2. ANOVA of the data of the third time bin (T3) failed to show statistically significant effects [*group*: F(1, 24) = 0.02, n.s.; *devaluation*: F(1, 24) = 0.68, n.s.; *devaluation* × *group*: F(1, 24) = 2.76, n.s.]. The analysis of the within-session time course of number of licks *per* burst of the second and third devaluation trials showed very similar results, with only the factor *devaluation* resulting in a statistically significant effect in all analyses, due to the reduced values observed in both groups in all time bins after reward devaluation [trial 2, within-session time course analysis: F(1, 17) = 49.28, P<10^−5^, T1: F(1, 37) = 62.65, P<10^−6^, T2: F(1, 28) = 31.41, P<10^−5^, T3: F(1, 21) = 11.24, P = 0.003; trial 3: within-session time course analysis: F(1, 20) = 37.40, P<10^−5^, T1: F(1, 37) = 62.53, P<10^−6^, T2: F(1, 32) = 21.33, P = 0.00006, T3: F(1, 22) = 5.06, P = 0.034] (Fig 4).

ANOVA of the intra-burst lick rate data showed a statistically significant effect of *devaluation* [F(1,37) = 6.58; P = 0.014], due to a slight increase of this parameter in response to the 2% sucrose solution, with no statistically significant effects of *trial* [F(2,74) = 0.27; n.s.] and *group* [F(1,37) = 2.33; n.s.] and no significant interaction between the three factors [*group* x *trial* x *devaluation*: F(2,74) = 1.08; n.s.] (Fig 3, bottom panels).

Since ANOVA of the latency to the first lick data failed to show any statistically significant main effect or relevant interactions [*group*: F(1,37) = 0.014, n.s.; *trial*: F(2,74) = 2.7, n.s.; *devaluation*: F(1,37) = 0.29, n.s.; interaction between the three factors: F(2,74) = 0.24; n.s.], we report the whole experiment mean value for each group: 14.95 ± 0.43 (G1) and 14.61 ± 0.45 (G2).

### Experiment 2

#### By sessions data analysis

**Fig 5:**
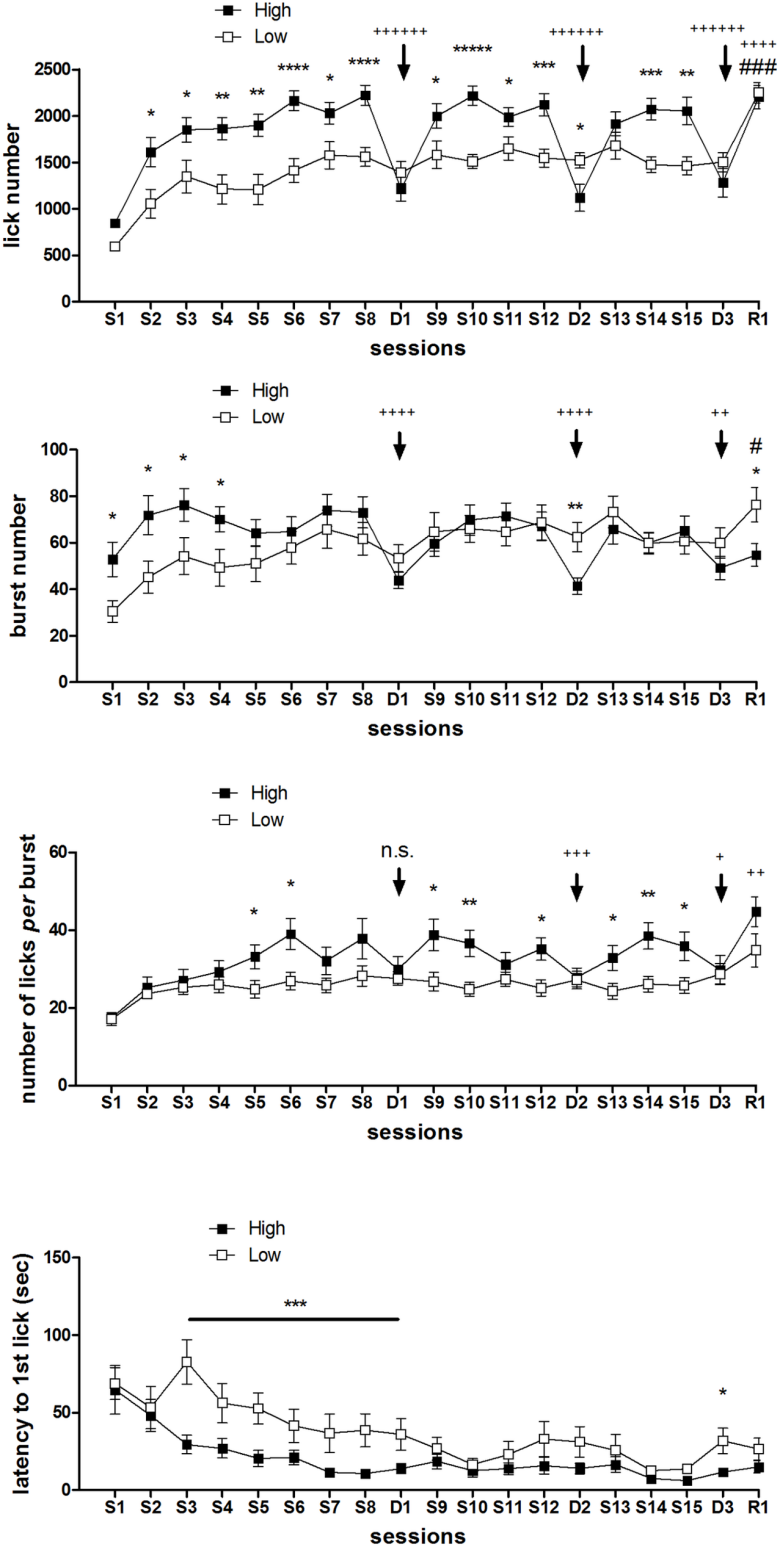
Experiment 2. **By sessions time course of lick number, burst number, number of licks *per* burst and latency to the first-lick (from top to bottom) in two groups of rats daily exposed to either a high (10%) or a low (2%) concentration sucrose solution.** In D1, D2 and D3 both groups were exposed to 2%, while in R1 to 10%. Values represent the mean ± S.E.M. from 16–19 subjects. Arrows indicate the devaluation episodes. High vs. Low concentration group: *P<0.05, **P<0.01, ***P<0.001, ****P<10^−4^, *****P<10^−5^, ******P<10^−6^. Within-subjects effect of devaluation, comparison between the devaluation sessions and the immediately preceding sessions in the High concentration group: +P<0.05, ++P<0.01, +++P<0.001, ++++P<10^−4^, ++++++P<10^−6^. Within-subjects effect of switch from 2% to 10%, comparison between R1 session and the immediately preceding session in the Low concentration group: #P<0.05, ###P<0.001 (ANOVA followed by F-test for contrasts).

ANOVA of lick number data (Fig 5, top panel) showed a statistically significant effect of *group* [F(1,33) = 10.53; P = 0.002] and *session* [F(18,594) = 21.10; P<10^−6^], with a statistically significant interaction between the two factors [F(18,594) = 6.06; P<10^−6^]. Further analysis (F-tests for contrasts) revealed that the high concentration group showed a higher lick number level, compared with the low concentration group, from the second session (S2) up till the end of the experiment. Moreover, a reduction in the lick number was observed in this group when exposed to the lower sucrose concentration in the three devaluation sessions (D1, D2, D3). Conversely, an increased lick number in the low concentration group was observed in the last session, when exposed to the 10% concentration (R1).

ANOVA of burst number data (Fig 5, 2^nd^ panel) showed a statistically significant effect of the factor *session* [F(18,594) = 5.03; P<10^−6^] but not *group* [F(1,33) = 0.35; n.s.], with a statistically significant interaction between the two [F(18,594) = 4.28; P<10^−6^]. The high concentration group showed higher levels of burst number in the first four sessions (S1-S4), with statistically significant decrements when exposed to the 2% concentration in the three devaluation sessions (D1, D2, D3). Conversely, an increased burst number in the low concentration group was observed in the last session, when exposed to the 10% concentration (R1).

As for both lick number and burst number, the values recorded in the devaluation sessions in the high concentration group were not significantly different from the values for the 2% solution in the low concentration group at sessions D1 and D3, while, at session D2, they dropped even to a lower level.

ANOVA of the whole session number of licks *per* burst data (Fig 5, 3^rd^ panel) revealed a statistically significant effect of the factors *group* [F(1,33) = 4.87; P = 0.03] and *session* [F(18,594) = 8.27; P<10^−6^], with a significant interaction between the two factors [F(18,594) = 2.27; P = 0.002]. F-tests for contrasts revealed a different response between the two groups starting at the 5^th^ session (S5): in the sessions from S5 to S15, in 8 time points out of 11, the high concentration group showed a significantly higher level of number of licks *per* burst. Exposure of the high concentration group to the 2% concentration resulted in a decrement of this measure in the 2^nd^ and 3^rd^ devaluation sessions (D2, D3), but not in the first one (D1). Exposure of the low concentration group to the 10% concentration (R1) failed to induce statistically significant effects.

**Fig 6:**
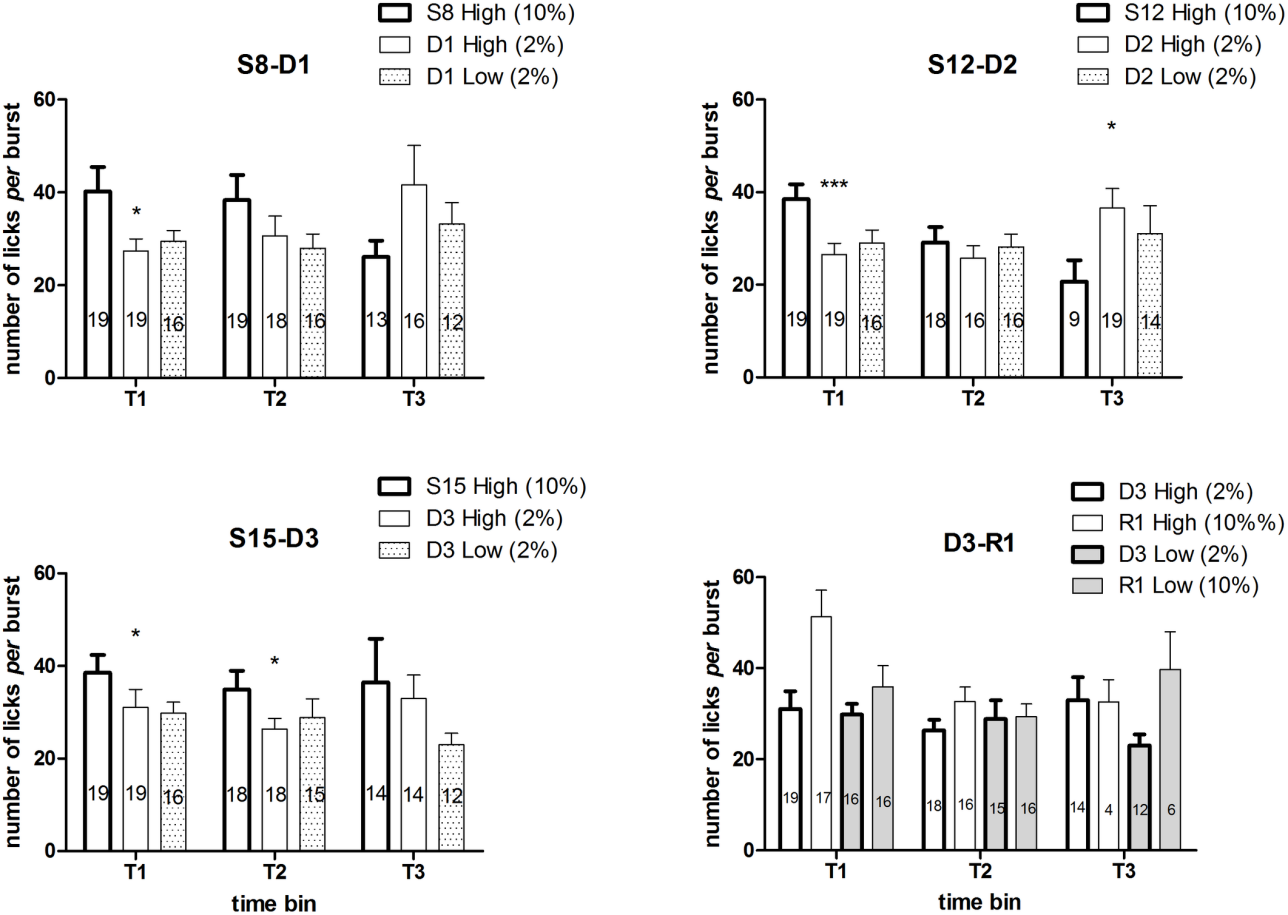
Experiment 2. **Effect of devaluation and of successive negative contrasts on number of licks *per* burst: within-session time course.** Values represent mean ± S.E.M. relative to (i) the session immediately preceding the devaluation session in the high concentration group (left column), and the (ii) high (central column) and (iii) low concentration group (right column) in the devaluation session. (i) vs (ii): devaluation effect; (ii) vs (iii): contrast effect. The number of subjects for each mean value is indicated in the relative column. Effect of *devaluation*: *P<0.05, ***P<0.001 (ANOVA, main effect).

ANOVA of the within-session time course of number of licks *per* burst of the first two devaluation trials in the high concentration group [S8 (10%) vs D1 (2%), S12 (10%) vs D2 (2%)] showed a significant interaction between the factors *devaluation* and *time* [S8 vs D1: F(2, 18) = 4.49, P = 0.026; S12 vs D2: F(2, 12) = 8.89, P = 0.0042], due to a reduction in burst size in the course of the session at S8 and S12 (exposure at 10%) compared to a specular increase at D1 and D2 (exposure at 2%). Consistently, the ANOVA of the single time bins showed a significant main effect of *devaluation* at T1 in both trials [S8 vs D1: F(1, 18) = 6.09, P = 0.02; S12 vs D2: F(1, 18) = 20.7, P = 0.00024], due to a reduction of burst size at D1 and D2, with no effect in the successive time bins in the trial S8-D1 [T2: F(1, 17) = 2.2, n.s.; T3: F(1, 10) = 2.2, n.s.], and a significant main effect in the third time bin (T3) in the trial S12-D2 [F(1, 8) = 7.9, P = 0.022], due to an increased burst size at D2. ANOVA of the within-session time course of number of licks *per* burst of the third devaluation trial in the high concentration group [S15 (10%) vs D3 (2%)] failed to show any statistically significant effects or interactions between the factors. Consistently with the first two trials, ANOVA of the single time bins showed a significant effect of *devaluation* in the first two time bins [T1: F(1, 18) = 4.89, P = 0.04; T2: F(1, 16) = 5.1, P = 0.03], due to a reduced burst size at D3, but not in the third [T3: F(1, 10) = 0.34, n.s.] (Fig 6).

ANOVA of the within-session time course of number of licks *per* burst of the first successive negative contrast (High concentration vs Low concentration group, both exposed to 2% at session D1) showed only a significant effect of *time* [F(2, 50) = 4.75, P = 0.012], due to the increased burst size in the last time bin (T3), regardless of group [*group* × *time*: F(2, 50) = 0.6, n.s.]. The analyses of the within-session time course of the number of licks *per* burst relative to the 2^nd^ (D2) and the 3^rd^ (D3) successive negative contrasts failed to show any statistically significant effect, as well as the ANOVA of the single time bins of all the three contrast sessions. (Here we report the results of the analysis relative to T3 at D3, which at visual inspection is the only comparison which might look like significant: F(1,24) = 2.83, P = 0.105, n.s.)(Fig 6).

ANOVA of the within-session time course of number of licks *per* burst of the upshift trial (High concentration and Low concentration group with 2% and 10% sucrose at sessions D3 and R1, respectively) showed only a significant effect of *upshift* [F(1, 5) = 22.22, P = 0.0052], due to the increased burst size after upshift from 2% to 10% sucrose concentration, regardless of *group* [*group* × *upshift*: F(1, 5) = 0.67, n.s.]. Moreover, a significant effect of *upshift* (increased burst size at session R1) was revealed in the first time bin [F(1, 31) = 9.002, P = 0.0052], again, with no interaction with the factor *group* [F(1, 31) = 2.59, n.s.]. No statistically significant effects or interactions were revealed by the analysis of the data relative to the successive time bins.

ANOVA of the intra-burst lick rate data failed to show a statistically significant effect of the factor *group* [F(1,33) = 3.86; n.s.], while a statistically significant effect of *session* was revealed [F(18,594) = 2.11; P = 0.004], due to slight differences between the sessions. The interaction between the two factors was not significant [F(18,594) = 0.43; n.s.](Table 2).

**Table 2:** Experiment 2. **Whole experiment mean intra-burst lick rate, and lowest and highest mean single session values from the High and the Low concentration groups.** Values represent the mean ± S.E.M. from 16–19 subjects.

Group	Whole experiment mean Intra-Burst Lick Rate	Lowest and highest mean single session value
High concentration	6.6±0.03	6.52±0.1–7.04±0.3
Low concentration	6.5±0.02	6.24±0.09–7.09±0.3

ANOVA of the latency to the 1^st^ lick data (Fig 5, bottom panel) showed a statistically significant effect of the factors *group* [F(1,33) = 8.56; P = 0.006] and *session* [F(18,594) = 10.17; P<10^−6^], with a significant interaction between the two [F(18,594) = 1.72; P = 0.03]. F-tests for contrasts revealed significantly lower latencies for the high concentration group from the 3^rd^ session (S3) up till the first devaluation session (D1). For the remaining part of the experiment no differences between the two groups were observed, with the exception of the session D3 (again, with the high concentration group showing a lower value).

#### Within-session analysis of burst number time course

**Fig 7:**
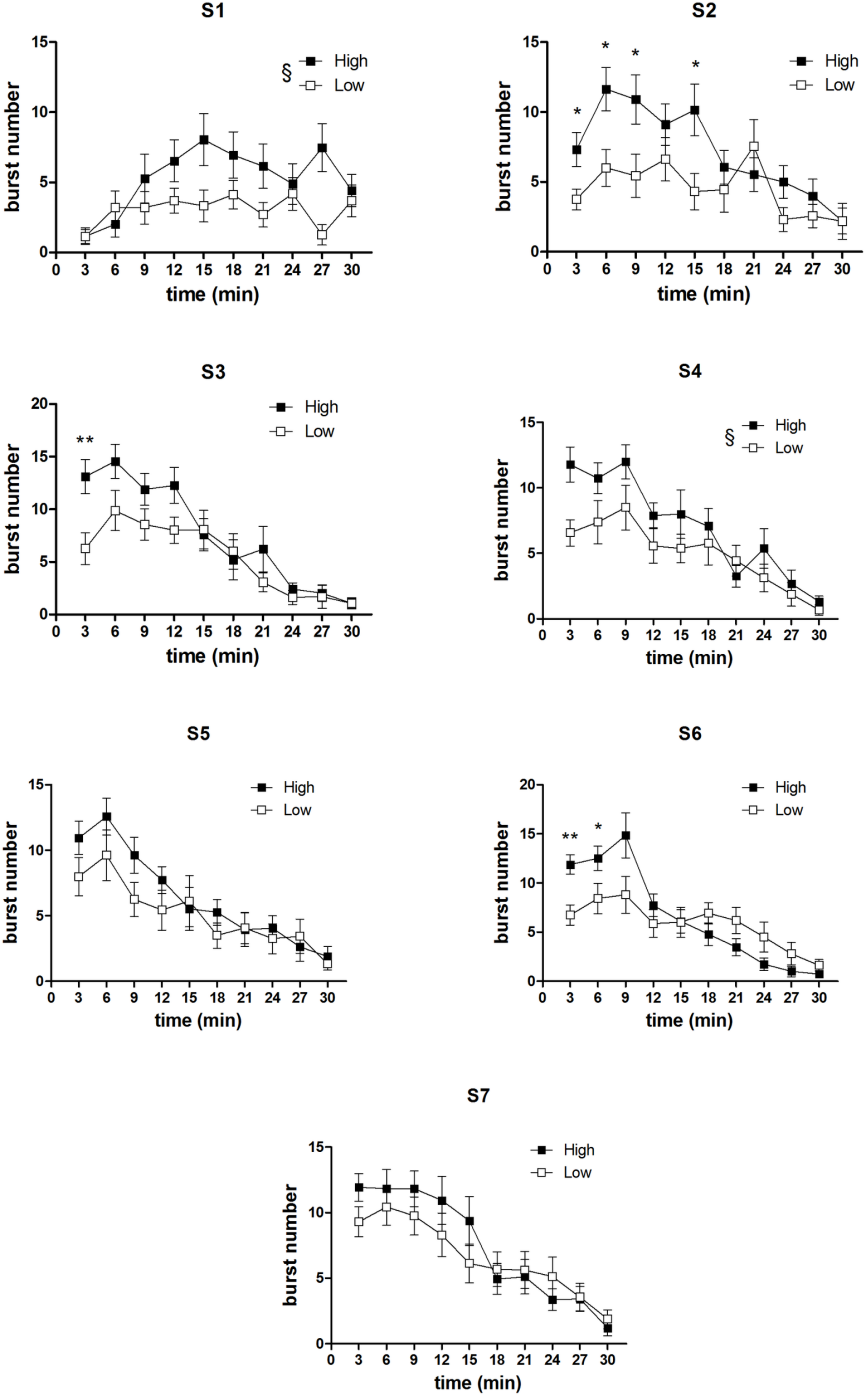
Experiment 2. **Within-session time course of burst number in the first seven sessions (S1-S7): comparison between the High and Low concentration groups.** Values represent the mean ± S.E.M. from 16–19 subjects. *P<0.05, **P<0.01 (Comparison between corresponding time bins, ANOVA followed by F-test for contrasts); §P<0.05 (ANOVA, main effect of *group*).

**Fig 8:**
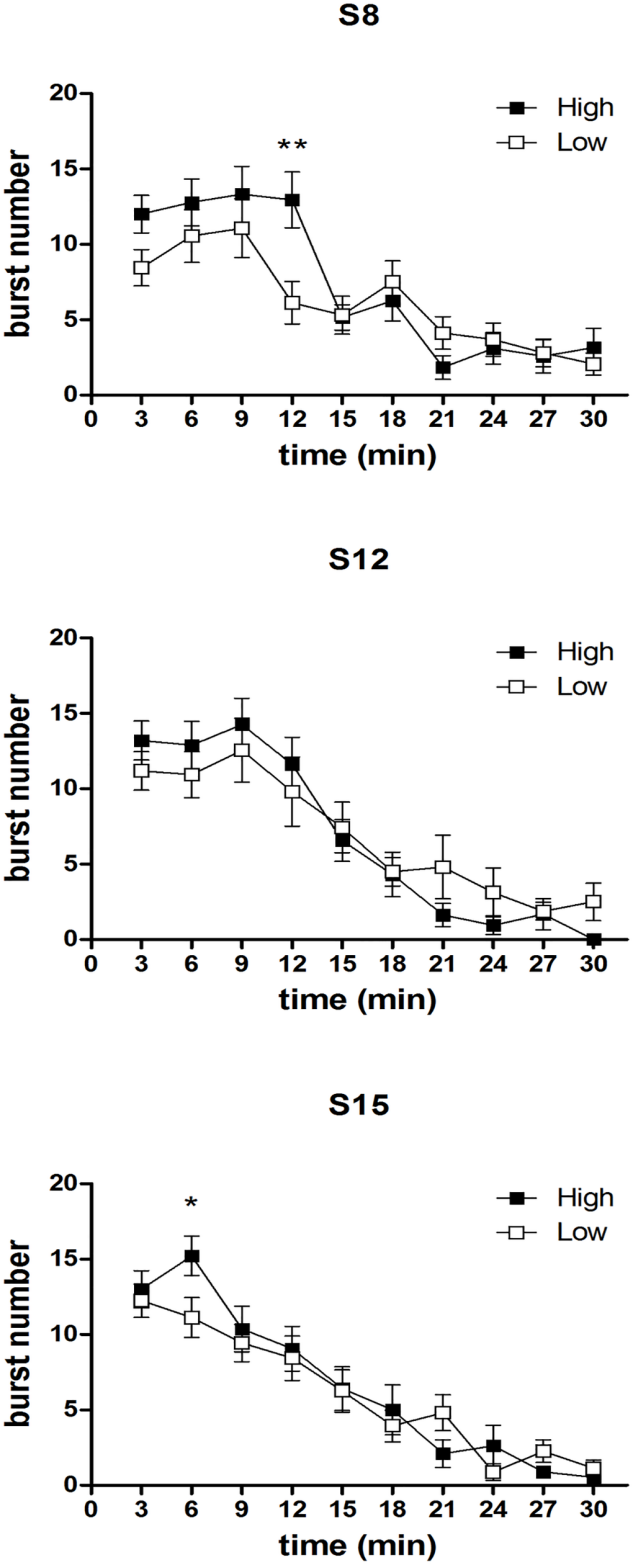
Experiment 2. **Within-session time course of burst number in the sessions immediately preceding the devaluation sessions (S8, S12, S15): comparison between the High and Low concentration groups.** Values represent the mean ± S.E.M. from 16–19 subjects. *P<0.05, **P<0.01 (ANOVA followed by F-test for contrasts).

In Fig 7 and Fig 8 are reported the comparisons between the burst number within-session time course from the two concentration groups, relative to all the sessions preceding the first devaluation episode and the single sessions immediately preceding the other two devaluation episodes. In the first four sessions (Fig 7, S1-S4), a statistically significant effect of the factor *group* was present [S1: F(1,33) = 6.04, P = 0.019; S2: F(1,33) = 5.81, P = 0.021; S3: F(1,33) = 4.37, P = 0.044; S4: F(1,33) = 4.84, P = 0.044], due to the higher level of burst number in the high concentration group. In the 2^nd^ and 3^rd^ sessions, an interaction between *group* and *time* was also present [S2: F(9,297) = 2.30, P = 0.16; S3: F(9,297) = 1.96, P = 0.04]. F-test for contrasts revealed that in both cases the difference between the two groups exposed to the different concentration solutions was apparent since the beginning of the session. In the other sessions examined (the sessions from S5 to S7, Fig 7, and the single sessions immediately preceding the devaluation sessions, i.e. S8, S12, S15, Fig 8) no effect of *group* was revealed [all F(1,33)<2.5, n.s.]. However, in three of these sessions (S6, S8, S15), a statistically significant interaction between the factors *group* and *time* (involving also the factor *devaluation* in S8 and S15 data) was revealed [S6: F(9,297) = 4.63, P = 0.000009; S8: F(9,297) = 3.47, P = 0.00042; S15: F(9,297) = 2.51, P = 0.0087]. In one case (S6), F-tests for contrasts revealed a statistically significant difference between the two groups in favour of the high concentration group, which was apparent since the beginning of the session and lasted for first six minutes (Fig 7). In the other two cases, the difference in favour of the high concentration group was limited to a single time bin (4^th^ time point for S8 and 2^nd^ time point for S15) (Fig 8).

**Fig 9:**
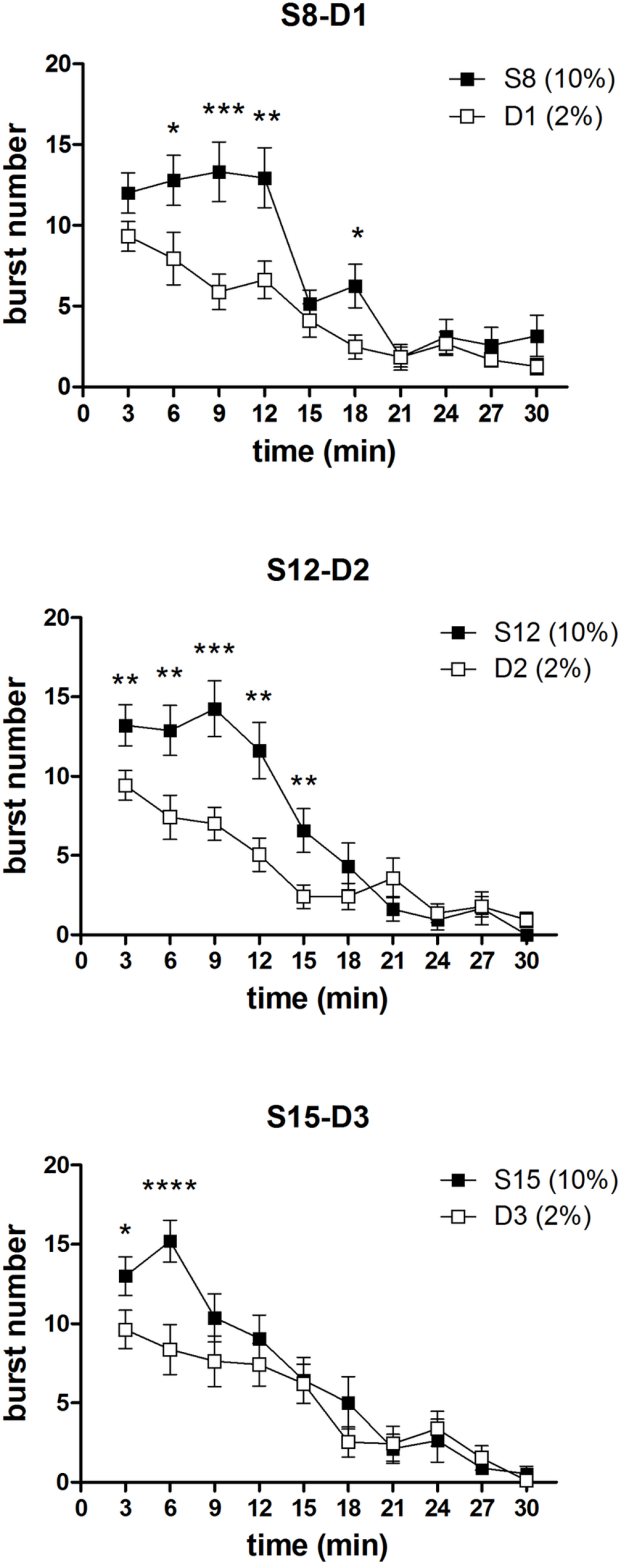
Experiment 2. **Within-session time course of burst number in the three devaluation trials (S8 vs D1, S12 vs D2, S15 vs D3) in the High concentration group.** Values represent the mean ± S.E.M. from 16–19 subjects. *P<0.05, **P<0.01, ***P<0.001 ****P<0.0001 (ANOVA followed by F-test for contrasts).

Fig 9 depicts the results of the devaluation trials (D1, D2, D3). In the high concentration group, the time course of the burst number response to a 2% concentration is compared to the time course of the immediately preceding session, with a 10% concentration. F-tests for contrasts, performed on the basis of a three ways interaction between the between-group factor *group* and the within-group factors *time* and *devaluation* [F(9,297) = 3.47; P = 0.004], have shown that, in the first devaluation episode, a statistically significant difference in favour of the 10% concentration is apparent only since the 2^nd^ time point, and persists up till the fourth time point, thus resembling the response pattern of an extinction curve, as observed in Experiment 1. In the successive devaluation episodes [*devaluation* x *time* x *group*: D2: F(9,297) = 2.47, P = 0.009; D3: F(9,297) = 2.51, P = 0.008], a significant difference is apparent since the beginning of the session, consistently with the observations from Experiment 1, showing an anticipated decrease of burst number after repeated devaluation episodes.

**Fig 10:**
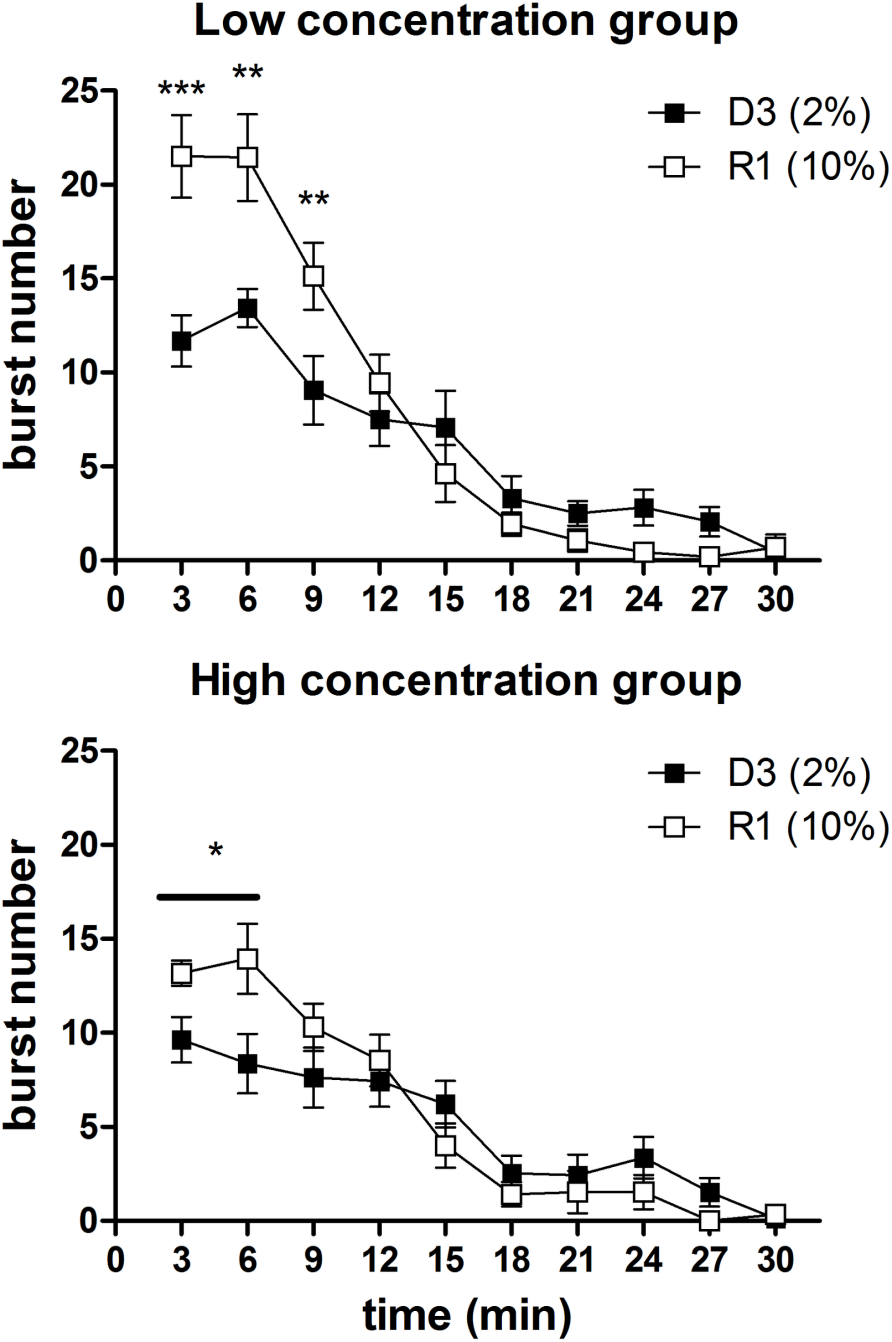
Experiment 2. **Within-session time course of burst number in the concentration upshift trial (D3 vs R1).** Values represent the mean ± S.E.M. from 16–19 subjects. *P<0.05, **P<0.01, ***P<0.001 (ANOVA followed by F-test for contrasts).

Fig 10 depicts the results of the upshift concentration trial (R1). The effect of the increase in sucrose concentration in the high and the low concentration group was very similar. Indeed, ANOVA showed a significant interaction between *time* and *upshift* [F(9, 279) = 10.19, P<10^−6^], with no three ways interaction between these factors and *group* [F(9, 279) = 1.22, n.s.], due to an increased burst number at the beginning of the R1 session,with respect to the preceding session (D3), in both groups. The lack of a significant interaction between *group* and *upshift* [F(1, 31) = 1.7, n.s.] indicates that the overall effect of the concentration upshift did not result in significant differences between the two groups. However, it should be recalled that in the whole experiment data analysis a statistically significant difference between the groups at session R1was revealed, based on the group × session interaction (see above, *By session data analysis*).

## Discussion

Consistently with earlier findings, the results of Experiment 1 showed that, in animals with daily access to a 10% sucrose solution, reward devaluation obtained by reducing the sucrose concentration solution to 2% resulted in a reduced ingestion, as indicated by the reduced lick number [see 8], mainly due to the reduction of the initial lick rate and of the whole session mean size of licking bursts, but also of their whole session number [2,3]. Moreover, both group G1 and group G2 responded to reward devaluation showing a within-session decrement of burst number, with the response rate decreasing only after contact with the devalued reward, which was observed in the first devaluation episode for G1 (trial 2), and in the first two devaluation episodes for G2 (trials 1 and 2). This response pattern was apparent also in the comparison between the response of group G2 to the 2% concentration and the response of group G1 to 10%, in the same experimental session (Fig 2: Trial 1 S.II). The results from Experiment 2 provided a replication of these findings. Indeed, all three devaluation sessions in the high concentration group (D1, D2, D3 compared to the immediately preceding session) resulted in a reduced lick number due to a reduction of the whole session size and number of licking bursts (with the exception of session D1, with a significant reduction of burst size observed in the 1^st^ 9-min time bin but not in the whole session data). Most importantly in relation to the aim of the present study, a within-session decrement of burst number occurring after the contact with the reward was observed in the first devaluation session (D1).

The most relevant comparison in relation to the aim of Experiment 2 was the comparison between the within-session burst number time course of the high *versus* low concentration group. We examined all the sessions preceding the 1^st^ devaluation episode, and the single sessions preceding the successive two devaluation episodes (Figs 6 and 7). In the first four sessions, when a significant difference in the session total burst number was present–which is the condition characterising all devaluation episodes–a difference in favour of the high concentration group emerged since the beginning of the session, with the possible exception of the first session. Indeed, the inspection of the data from such session shows both groups with very low burst number levels at the beginning of the session, as one would expect in subjects at their first experience with the apparatus, with the high concentration group increasing more steeply and eventually reaching a higher level, as one would expect with a larger reward. However, these within-session differences were not supported by a statistically significant interaction between the relevant factors (*group* and *time*). When the difference in total values was not present, the within-session burst number time courses of the high and of the low concentration group were virtually super-imposable. (With the exception of three sessions: in one session [S6], a significant difference in favour of the high concentration group was present, again, since the beginning of the session, and lasted for six minutes; in the other two sessions [S8 and S15], it was limited to a single time point.) Hence, these results show that the difference in the burst number within-session time course in two groups of subjects in response to two different sucrose concentrations, i.e. in a condition which does not involve changes in the reward value, is very different from the extinction pattern observed with reward devaluation.

Thus, the results from this study, taken together with the results of our previous studies on the effect of dopamine D1- and D2-like receptor antagonists on licking microstructure [8, 9], provide evidence confirming the proposed interpretation: the within-session decrement of the burst-number time course occurring after the contact with the reward, observed with dopamine D2-like receptor blockade, can be interpreted as an extinction-like effect, in that it resembles the response pattern observed after reward devaluation. Moreover, dopamine D1-like receptor blockade, which results in a lower level of this measure apparent since the beginning of the session, reproduces the difference observed comparing the response to either a high or a low sucrose concentration solution in separate groups of rats, i.e. in a condition which does not involve changes in the reward value, but yields different levels of behavioural activation. It should be stressed that while the present data have important implications for the interpretation of the results of our previous studies on the role of dopamine in behavioural activation through the analysis of licking microstructure, the investigation of the role of dopamine–as well as of any other biochemical substrate or neural system–in the ingestive behavioural responses to shifts in the reward value, such as the responses described in the present study, requires further studies involving experimental manipulations of the relevant neural substrates.

In both experiments (see Figs 2 and 9), re-exposure to sucrose dilution after just one or two devaluation episodes failed to produce a clear within-session extinction pattern, but resulted in a decrease of burst number since the beginning of the session (Exp. 1, both groups: trial 3; Exp. 2, High concentration group: S12 vs D2, S15 vs D3). Such an anticipated response might depend on a learning process leading to a more rapid update of the level of activation of the emission of licking bursts in response to reward devaluation. According to this interpretation, in the first devaluation episode, the licking burst emission response undergoes (partial) extinction: it starts as a response which is appropriate for (and was reinforced by) the previous contacts with the 10% solution; following the contact with the devalued reward (2% solution), its level of activation is reduced to that which is appropriate for 2%. Due to the learning process, in the successive contact with the devalued reward, the switch from the 10% to the 2% appropriate response activation level is more rapid, leading to a low burst number level since the beginning of the session. However, experiments specifically designed to the scope are necessary to test this interpretation.

In Experiment 1 (Fig 4), the investigation of the effect of reward devaluation on burst size at different times within the session, showed a reduction of this measure in all three time bins, with the exception of trial 1 for group G2, with a lack of a statistically significant effect in the 3^rd^ time bin. In Experiment 2, burst size reduction was consistently observed in the 3 devaluation trials (S8 vs D1, S12 vs D2, S15 vs D3 in the High concentration group) only in the first time bin (at session D3 it was present also in the 2^nd^ time bin). In the 3^rd^ time bin of session D2, burst size was increased. Thus, the results from Experiment 2 might suggest a within-session attenuation of the effect of reward devaluation on burst size. Such an effect appears to depend (i) to the trend in decrease of burst size over time in the session preceding devaluation, when the animals are exposed to the familiar concentration (10%), and (ii) to a specular increase in burst size in the devaluation session (these patterns are apparent in the first two devaluation sessions). However, considering the lack of full consistency between the results from the two experiments, no conclusions can be drawn on this matter on the basis of the present set of data, which simply shows that, in our experimental conditions, the effect of reward devaluation of reducing burst size is more reliably observed early in the session.

The comparison between the whole session data of the response of the high *versus* low concentration group to two different sucrose concentrations, 10% vs 2%, respectively (Experiment 2, Fig 5), showed significant differences since the first session, in animals at their first experience with the sweet solutions, with a higher burst number, but not burst size, in the high concentration group. The difference in lick number, which is proportional to ingested volume [8], with higher levels in the high concentration group, emerged in the second session and lasted up to the end of the experiment, and it was accounted for by a difference in burst number up to the 4^th^ session, and by a difference mainly in burst size from the 5^th^ session on. Both groups showed a progressive increase in burst number across sessions, with a more rapid increase in the high concentration group, and with both groups eventually reaching the same (relatively stable) level after a week. The time course of the latency to the 1^st^ lick values across sessions showed both groups at the same level in the 1^st^ two sessions. The high concentration group values progressively decreased across the first week sessions, reaching a low value which remained stable up to the end of the experiment. The decline of the values of the low concentration group was less steep, reaching the level of the high concentration group from the 9^th^ session up to the end of the experiment (with the exception of session D3, with the low concentration group showing a slight but statistically significant increase with respect to the high concentration group). The difference between the two groups in the speed of the progressive increase in burst number across sessions, and of the almost specular progressive reduction of latency to the 1^st^ lick, reflects the different reinforcing effect of the two sucrose concentrations. Thus, these results show that the response to reinforcement of burst number across sessions is similar to that of the response latency, which, in a number of experimental paradigms, is considered as a measure of behavioural activation [6, 31, 32].

The increased burst size observed from the 5^th^ session in the high concentration group is due to a progressive increase of this parameter across sessions, which might depend, at least in part, by an habituation process, possibly consisting in a gradual overcome of the inhibitory feed-back provided by novelty of taste [33]. The higher burst size was crucial in leading to the higher overall intake (lick number) in this group. These results also suggest that burst number might reflect the effect of the evaluation process occurring during the consummatory transaction with the reward more promptly than burst size.

The comparison between the whole session data of the response of the high *versus* low concentration group to the low sucrose concentration solution (Experiment 2, D1, D2, D3. Fig 5) revealed the successive negative contrast effect only in session D2: indeed, the intake of the 2% solution, revealed by the lick number, was reduced in the high concentration group, subjected to down-shift in sucrose concentration, with respect to the “unshifted” low concentration group. This reduction was entirely due to a lower level of burst number. No differences in burst size between the two groups were found (Fig 5, whole session, and Fig 6, time bins). The failure to observe the contrast effect more robustly might depend on the sucrose concentrations used in our experiments. Indeed, most experiments on successive negative contrast with sucrose use 32% as the high concentration and 4% as the low concentration [29, 30], or 1M vs 0.1M [28], alternatively. With these concentrations one obtains a higher degree of dilution (1/8-1/10) compared to our experiment (1/5), providing a stronger reward devaluation, and a more concentrated low concentration solution, which might support a higher response level in the low concentration group.

The results of the upshift trial showed that exposure to the 10% solution in R1 yielded an increased lick number both in the high and in the low concentration group, with respect to the previous session with 2% (D3), mainly due to increased whole session burst size in the high concentration group and to increased whole session burst number in the low concentration group. The analysis of the results of the single time bins suggests that the increase in burst size after the concentration upshift is accounted for by the response early in the session. The analysis of the within-session time course of burst number showed a similar response pattern for both groups, characterised by an increased response at the beginning of R1 session with respect to D3. The comparison between the two groups at session R1 showed a higher burst number in the low concentration group, paralleled by a lower (but the difference was not statistically significant) burst size value, with the result that lick number did not differ between the two groups. These results show that, while responding to an increase in the reward value, the level of emission of licking bursts is immediately updated to the level which is appropriate for the increased value reward. This is the opposite with respect to devaluation, with the response persisting for several minutes before dropping to a lower level. Thus, these observations provide support to the interpretation of burst number as a measure of behavioural activation. Moreover, this makes sense in adaptive terms. Indeed, it might be advantageous to persist in a response which is not particularly demanding in terms of effort while dealing with a devalued reward, since small reductions in the reward value might be compensated by an increased response rate [19], while there is no possible gain in persisting with a low response rate while dealing with a reward with an increased value.

In Experiment 1, we observed a slight increase in the intra-burst lick rate after reward devaluation, while in Experiment 2 we observed slight differences in this measure between sessions (regardless of group). This might seem somewhat surprising. Indeed, the rhythm of the tongue movements while licking is extremely stable, depending on a brainstem Central Pattern Generator (CPG) the activity of which can occur without proprioceptive feedback and without descending input from the cortex [34]. However, very small changes of this parameter had been previously observed in response to experimental manipulations not involving drugs (which can have direct effects on CPG neurons). For example, in rats licking for NaCl solutions, the intra-burst lick rate was influenced by NaCl concentration and by sodium-depletion [23]. It might be worth noting that such effects are very small and have, if any, negligible effects on intake.

The results of this study might provide further elements for the interpretation of the effects of either drugs or other experimental manipulations on ingestive behaviour. Based on the lack of effect on burst size and initial lick rate, two previous studies reported the failure to observe anhedonic effects after administration either of the dopamine D2-like receptor antagonist eticlopride [35] or of the opioid antagonist naltrexone [36]. However, a within-session decrement of lick number was observed in both studies, which, in absence of effects on burst size, can be explained only by a within-session burst number time course with a similar pattern. The reduction of burst size and initial lick rate are considered as signs revealing anhedonia because these effects were observed after sucrose dilution, i.e. after reduction of the reward value [2–4, 7]. Here we provide evidence that there is another distinctive feature which characterises the licking response to reward devaluation: the within-session decrement of burst number after the contact with the devalued reward. Incidentally, an independent replication of our previous finding on the effect of dopamine D2-like receptor blockade on licking microstructure might be provided by one of the two cited studies, which was performed in a different species (mice), with a different dopamine D2-like receptor antagonist (eticlopride) and a different route of administration (lateral ventricle infusion)[35].

In conclusion, these results show that the analysis of burst number, but not of burst size, reveals a specific activation pattern in response to reward devaluation, which differs from the pattern observed comparing the response to two different sucrose concentrations in separate groups of subjects. These response patterns are paralleled by similar ones induced by either D2-like (extinction mimicry) or D1-like (different activation level) receptor antagonism [8, 9]. Thus, these observations might bear relevance in relation to the ongoing debate on the role of dopamine on goal-directed behaviour [3, 6, 8, 9, 17, 19, 20, 23–25, 35–39]. Moreover, this contribution to the characterisation of the analysis of licking microstructure might help to better define the experimental measures in behaviourally (and psychologically) meaningful functional terms. Here we provide evidence in support of the interpretation of burst number as a measure of behavioural activation, whose analysis might reveal the evaluation process determining its emission level. Finally, these findings suggest that when analysing the effects of either drugs or taste manipulations on licking microstructure, the within-session time course of this measure should be taken into account, and provide further support to the analysis of licking microstructure as a potentially important source of behavioural substrates, relevant for the investigation of the mechanisms underlying behavioural activation and the related evaluation processes.
